# Creation and characterization of novel rat model for recessive dystrophic epidermolysis bullosa: Frameshift mutation of the *Col7a1* gene leads to severe blistered phenotype

**DOI:** 10.1371/journal.pone.0302991

**Published:** 2024-05-09

**Authors:** William Stone, Chloe Strege, William Miller, Aron M. Geurts, Michael Grzybowski, Megan Riddle, Christopher Lees, Cindy Eide, Douglas R. Keene, Sara F. Tufa, Davis Seelig, John McGrath, Jakub Tolar

**Affiliations:** 1 Department of Pediatrics, Medical School, University of Minnesota, Minneapolis, Minnesota, United States of America; 2 Department of Physiology, Medical College of Wisconsin, Milwaukee, Wisconsin, United States of America; 3 Research Department, Shriners Hospital for Children, Portland, Oregon, United States of America; 4 Comparative Pathology Shared Resource, College of Veterinary Medicine, University of Minnesota, Minneapolis, Minnesota, United States of America; 5 St. John’s Institute of Dermatology, King’s College London (Guy’s Campus), London, United Kingdom; University of Colorado Boulder, UNITED STATES

## Abstract

Recessive dystrophic epidermolysis bullosa is a rare genodermatosis caused by a mutation of the *Col7a1* gene. The *Col7a1* gene codes for collagen type VII protein, a major component of anchoring fibrils. Mutations of the *Col7a1* gene can cause aberrant collagen type VII formation, causing an associated lack or absence of anchoring fibrils. This presents clinically as chronic blistering, scarring, and fibrosis, often leading to the development of cutaneous squamous cell carcinoma. Patients also experience persistent pain and pruritus. Pain management and supportive bandaging remain the primary treatment options. The pathology of recessive dystrophic epidermolysis bullosa was first described in the 1980s, and there has since been a multitude of encouraging treatment options developed. However, *in vivo* research has been hindered by inadequate models of the disease. The various mouse models in existence possess longevity and surface area constraints, or do not adequately model a normal human disease state. In this paper, we describe a novel rat model of recessive dystrophic epidermolysis bullosa that offers an alternative to previous murine models. An 8-base pair deletion was induced in the *Col7a1* gene of Lewis rats, which was subsequently found to cause a premature stop codon downstream. Homozygous mutants presented with a fragile and chronically blistered phenotype postnatally. Further histological analysis revealed subepidermal clefting and the absence of anchoring fibrils. The generation of this novel model offers researchers an easily maintained organism that possesses a larger surface area for experimental topical and transfused therapies to be tested, which may provide great utility in the future study of this debilitating disease.

## Introduction

Recessive dystrophic epidermolysis bullosa (RDEB) is a rare inherited blistering skin disease [1]. Minor trauma can result in major fragility of the skin and some mucous membranes which can lead to wounds that are slow to heal and become chronic. Wound healing is associated with fibrosis and contractures. Chronic skin inflammation and scarring can be complicated by the development of cutaneous squamous cell carcinomas (cSCC) [2]. There is also systemic inflammation and extracutaneous co-morbidities such as anemia and osteopenia [2]. Major symptoms include pain and itch. Clinically, the severity of the blistering and scarring in RDEB may vary, which is mostly explained by the underlying molecular pathology. RDEB results from bi-allelic pathogenic variants in the *Col7a1* gene, which encodes type VII collagen (C7). These proteins comprise a major component of anchoring fibrils, which form key attachment complexes that secure the adhesion of the epidermis to the underlying dermis [3]. *Col7a1* gene variants (combinations of missense, nonsense, indel, splice site) lead to a reduction or complete absence of C7, which results in either a reduced number of poorly functional anchoring fibrils or a complete lack thereof.

In recent years, considerable efforts have been made to develop new forms of gene, cell, protein, and small molecule therapies, although current best clinical practice mostly just offers supportive care without cure [4]. Critical to all therapeutic developments is the need to develop suitable animal models that reflect the RDEB phenotype, its clinical course, and its disease complications. Naturally occurring RDEB has been described in dogs, cats, cattle, sheep, goats, and ostriches [5], although only one dog model is proving to have value in RDEB therapy testing [6]. With regards to engineered models of RDEB, most are murine.

While researchers are constantly trying to improve established models, studying RDEB continues to be difficult given the recessive nature of the disease, as well as the limitations of size and survivability of current *in vivo* research models [7–9]. While *in vitro* work is valuable for gathering initial insights, it fails to replicate the dynamic environment of whole organisms. Limitations of *in vitro* research have prompted the development of a multitude of RDEB mouse models. Initially, *Col7a1* knock-out (KO) mouse models were developed. However, the resulting phenotype of these animals is severe, limiting the survivability of *Col7a1*^*-/-*^ pups and therefore their effective use in research applications [7]. In hopes of improving the survivability of these animals, *Col7a1*^*-/-*^ models were developed on an immunocompromised NSG base mouse strain [8]. The affected pups had improved survivability, but the absence of an immune response does not accurately recapitulate a human disease state. To improve survivability without removing important factors in studying the disease, a hypomorphic model was created using a gene cassette insertion method. This model presents with a phenotype typical of other murine RDEB models, but with minimally improved survivability due to having about 10% gene expression of *Col7a1* [9]. However, this variation in expression does not genetically resemble any RDEB mutational status observed in human patients, and long-term survivability remains lacking. Although these models have proved useful in studying RDEB, the search for improved models persists.

Rats have historically been selected as model organisms for testing innovative therapies, but their use has dwindled, in part due to transgenic technologies being more developed and efficient for creating KO mice [10]. In this study, we aimed to improve upon previously established RDEB murine models by utilizing Lewis rats (Lew/Crl) as a novel model of the disease. We hypothesized that using rats to model RDEB could help to ameliorate constraints observed in previous mouse models, such as limited surface area and survivability of the diseased pups. The creation and characterization of this RDEB rat model provides an alternative model for studying RDEB with the hope of advancing this field of research.

## Methods

### Murine care and characterization

Research was approved by the University of Minnesota Institutional Animal Care and Use Committee under protocol 2106-39156A and was carried out in strict accordance with the recommendations for the ethical treatment and care with all efforts made to minimize suffering. Lewis rats were kept on a 12-hour light:dark cycle, given food and water *ad libitum*, and housed in temperatures between 22–24°C. Daily health checks were performed, and litter sizes and survivorship of *Col7a1*^*del8/del8*^ pups were recorded during that time. Since EB symptoms vary dramatically between animals, endpoints were dealt with on a case-by-case basis. The following humane endpoints resulted in immediate euthanasia: 20% weight loss, inability to reach food or water, not nursing in the case of pre-weaned rats, and failure to grow. Survival analysis of *Col7a1*^*del8/del8*^ was performed in GraphPad Prism v 9.4.1 (458), and the date of birth was defined as day 1. Wild-type (WT) littermates were used for experimental comparisons.

### Immunofluorescence staining

RDEB rat and WT skin tissue samples were frozen in optimal cutting temperature (OCT, Sakura Finetek USA, Torrance, CA) and sectioned at 6 microns on a cryostat. Sections were fixed in room temperature acetone for 5 minutes. Tissue sections were blocked with 10% normal donkey serum for 1 hour (Jackson Immunoresearch, West Grove, PA). Primary antibody C7 (1:200 LifeSpan BioSciences, Seattle, WA) was incubated overnight at 4°C. Slides were washed with 1× PBS. Secondary donkey anti-rabbit Cy3 (Jackson Immunoresearch, 1:500) was applied for 1 hour at room temperature. Slides were washed with 1× PBS and then cover-slipped with hard-set DAPI, 4,6-diamidino-2-phenylindole (Vector Labs, Burlingame, CA). Slides were examined by confocal fluorescence microscopy (Olympus BX61, Olympus Optical, Tokyo, Japan).

### Histopathology analysis

Harvested tissue was fixed in 10% neutral buffered formalin at 4°C for 24 hours. Tissues were then washed 3× with Dulbecco’s phosphate-buffered saline and stored in 70% ethyl alcohol at 4°C until they were paraffin-embedded, sectioned, mounted onto slides, and stained with hematoxylin/eosin (H&E). Interpretation of histological findings was performed by veterinary pathologists within the Comparative Pathology Shared Resource at the University of Minnesota.

### Transmission electron microscopy

Skin from the belly and footpad of one-day-old WT and *Col7a1*^*del8/del8*^ rats was submersed overnight in either 1.5% glutaraldehyde/1.5% formaldehyde with 0.05% tannic acid or Dulbecco’s Modification of Eagle’s Medium (DMEM). For immuno-electron microscopy, samples stored in DMEM were immersed in rabbit anti-Col VII antibody (LifeSpan BioSciences cat LS-C294080/226005) diluted 1:20 in DMEM, rinsed, then in goat anti-rabbit ultrasmall gold conjugate (Aurion Biotech, Seattle, WA) diluted 1:3 in DMEM, rinsed again, then immersed in gold enhancement solution (Nanoprobes.com, Long Island, NY). Tissues were then fixed in 1.5% glutaraldehyde/1.5% formaldehyde with 0.05% tannic acid. All tissues were post-fixed in 1% osmium tetroxide, dehydrated in a graded ethanol series to 100%, exposed to propylene oxide, then infiltrated in Spurrs’ epoxy and embedded. 80nm ultrathin sections were stained in uranyl acetate and lead citrate, then photographed using an AMT 2×2K camera on a FEI G2 TEM operated at 120 KV.

### CRISPR specific gene targeting

Lewis rats (Lew/Crl) were chosen as the model organism for gene-targeted CRISPR injections. Target region fell within exon 1 of the *Col7a1* gene (target sequence: GCACTGCCGAGATCCTGGTGGGG). A CRISPR single guide RNA was introduced along with SpCas9 RNP via pro-nuclear injection into one-cell embryos. Founder animals were screened using PCR-fluorescent fragment-length analysis for the target locus using Applied Biosystems^™^ 3730xl capillary sequencer. Mutational accuracy was verified using Sanger sequencing. Once confirmed, founders were backcrossed with parental strain to establish a stable genetic line. Primers used: 5’-TGGGGAACACAGAGTAGAATTCAAGG-3’ and 5’-AGGCAAGATTAGGAAGGACTTGGGG-3’.

### Sequencing of mutagenic region

Tail snips were obtained at time of weaning, and DNA was isolated from this tissue using the PureLink^®^ Genomic DNA Mini Kit (Cat# K1820-02). A 339-base pair (bp) *Col7a1* PCR product was generated and purified using NucleoSpin^®^ Gel and PCR Clean-up (Cat# 740609.50). Primers used: C7 Fwd (5’-TGGGGAACACAGAGTAGAATTCAAGG-3’) and C7 Rev (5’-AGGCAAGATTAGGAAGGACTTGGGG-3’). Purified PCR product was then sent to Sequetech^™^ for Sanger sequencing with the previously mentioned primers. The resulting sequences were then compared using ApE—A plasmid Editor (v 3.0.9) software.

## Results

### Phenotype characterization

**Fig 1:**
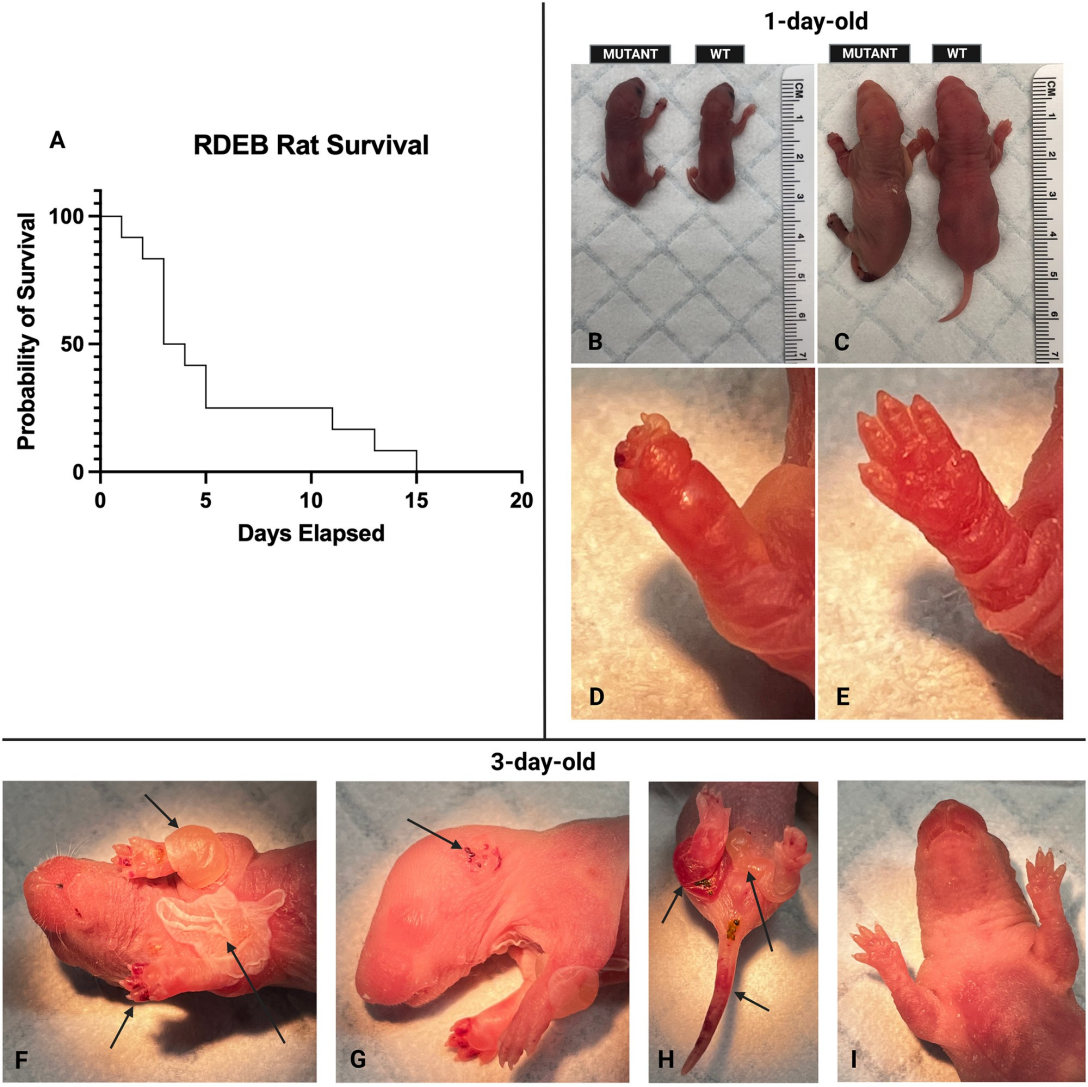
Characterization of *Col7a1*^*del8/del8*^ rat phenotype. **A)** Kaplan-Meier survivorship curve (n = 12) of *Col7a1*^*del8/del8*^ pups. Median survival = 3.5 days, mean survival = 5.7 days. **B-C)** Prone size comparison between **B)** *Col7a1*^*flNeo/Neo*^ and WT hypomorphic mouse pups [9] and **C)** *Col7a1*^*del8/del8*^ and WT rat pups. **D)** Paw of *Col7a1*^*del8/del8*^ pup presenting with pseudosyndactyly, and **E)** WT littermate. **F)** *Col7a1*^*del8/del8*^ pup in supine position displaying both intact and burst nonhemorrhagic blistering. Arrows indicate locations of cutaneous blistering at various stages of healing. **G)** Right lateral recumbency of *Col7a1*^*del8/del8*^ pup with blistering of ear. **H)** *Col7a1*^*del8/del8*^ pup with cutaneous blistering to posterior. Arrows indicate the affected areas on the anatomical right-rear limb, tail and belly. **I**) WT rat pup littermate.

Spontaneous hemorrhagic blistering of *Col7a1*^*del8/del8*^ pups occurred shortly after birth (Fig 1B–1E). While there was slight variation in the severity and location of blisters, the blistering of footpads, often extending into the upper limbs, was consistently observed on all affected pups. After 2–4 days, nonhemorrhagic blisters developed on the upper trunk, neck, or belly of pups (Fig 1F–1I). There were observations of minor blistering to the mouth, pinnae, and nares of affected pups. Pups that were able to live past 7 days presented with whole-limb swelling and inflammation. Sloughing of skin was observed in *Col7a1*^*del8/del8*^ pups. The median survival of *Col7a1*^*del8/del8*^ pups was characterized to be 3 days (Fig 1A).

### Immunofluorescence and H&E staining

**Fig 2:**
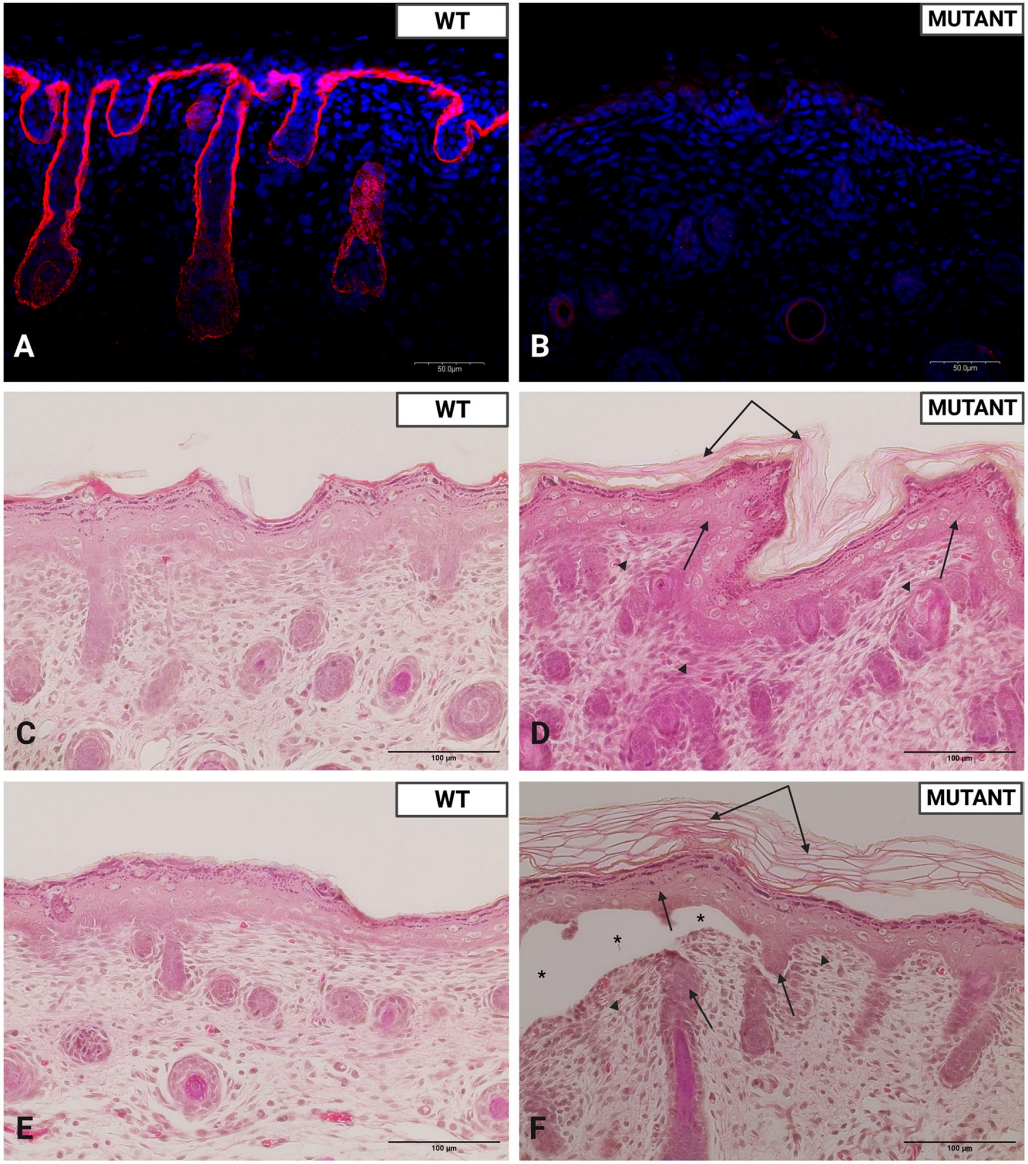
Immunofluorescence and H&E staining of RDEB rat skin, demonstrating respective aberrant collagen type VII formation and blistering within the DEJ. **A-B)** Rat WT and *Col7a1*^*del8/del8*^ skin costained with LSbio collagen VII antibody (red) and DAPI (blue) at 40× magnification; scale bars = 50 μm. **C-F)** H&E staining of skin from one-day-old WT and *Col7a1*^*del8/del8*^ rat pups at 40× magnification; scale bars = 100 μm. Double arrows indicate areas of hyperkeratosis observed. Areas of mild multifocal epidermal hyperplasia are noted with single arrows. Subepidermal clefting was observed in *Col7a1*^*del8/del8*^ samples (*). Arrowheads point to areas of mild multifocal hypercellularity of superficial dermis.

Immunofluorescent staining of WT and *Col7a1*^*del8/del8*^ skin demonstrated more C7 present in WT versus *Col7a1*^*del8/del8*^ skin (Fig 2A and 2B). Localization of C7 staining in WT mice was observed in dermal-epidermal junction, consistent with the basement membrane zone, and in localized skin adnexal structures. Minimal diffuse staining was noted in *Col7a1*^*del8/del8*^ skin in areas mentioned above. No background staining was noticed in either group.

H&E staining revealed the following notable differences between one-day-old *Col7a1*^*del8/del8*^ and WT skin: 1) Subepidermal clefting. 2) Mild multifocal epidermal hyperplasia presenting as a slightly thickened epidermis and more prominent rete pegs (Fig 2C–2F). 3) Hyperkeratosis. 4) Mild multifocal hypercellularity of the superficial dermis.

### Transmission electron microscopy

**Fig 3:**
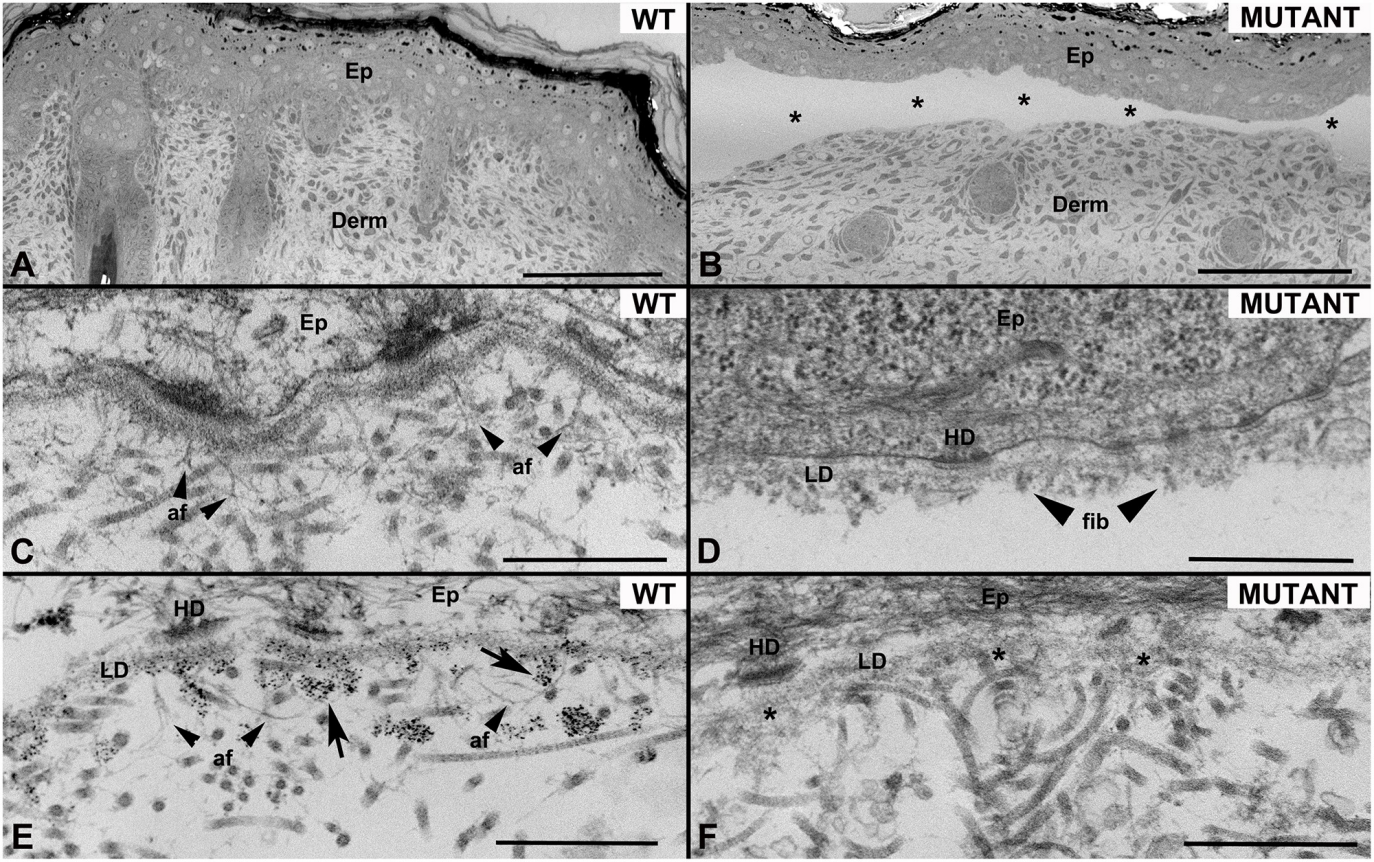
Transmission electron microscopy of WT and *Col7a1*^*del8/del8*^ skin. Low magnification demonstrating **A)** intact WT skin and **B)** full-thickness separation (*) of epithelium (Ep) from dermis (Derm) in *Col7a1*^*del8/del8*^ skin. Distinct arching and looping anchoring fibrils (af, arrows) extend into the dermis and entrap banded collagen fibrils in **C)** WT skin but are absent in **D)** *Col7a1*^*del8/del8*^ skin. The plane of separation in *Col7a1*^*del8/del8*^ skin is deep to the lamina densa (LD), which is thinner and less dense than in WT. Fibrous material (fib, arrows) is associated with the LD in **D)** *Col7a1*^*del8/del8*^ skin. **E)** Immuno-gold labeling (arrows) for type VII collagen is strongly positive in WT skin at the lamina densa (LD), with most intense labeling below hemidesmosomes (HD) where anchoring fibrils (af, arrowheads) are concentrated. **F)** Type VII collagen labeling was absent in *Col7a1*^*del8/del8*^ skin. The sub-epithelial LD is poorly defined in intact regions due to accumulation of fibrous material (*). Scale bars: A,B = 100 microns; C-F = 500 nm.

Transmission electron microscopy (TEM) of WT rat skin and footpad showed fully intact tissue (Fig 3A–3F). The basement membrane zone was dense with thin, arching, and looping anchoring fibrils. Antibody labeling was most intense at anchoring plaques. Anchoring fibrils were occasionally banded or branching. Comparatively, *Col7a1*^*del8/del8*^ skin and paw revealed an absence of anchoring fibrils with no C7 antibody labeling present. The lamina densa was wispy and less dense than WT tissue, and large or full-thickness sublamina densa splits were observed. Aggregation of fibrous material was also associated with the dermal-epidermal junction of *Col7a1*^*del8/del8*^ tissue.

### Mutation characterization

**Fig 4:**
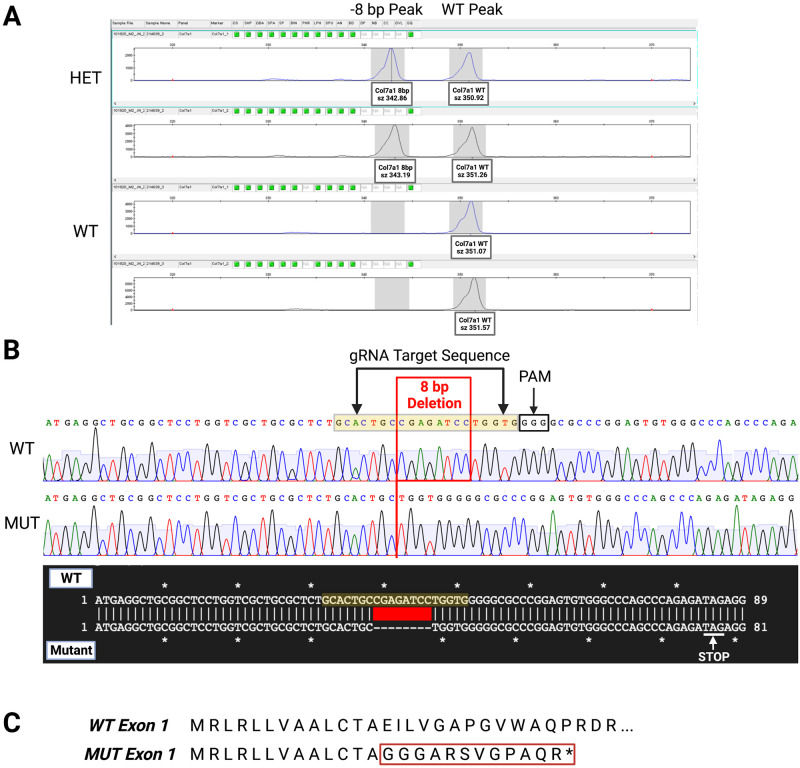
Characterization of the CRISPR-Cas9 induced mutagenic region. **A)** PCR-fluorescent fragment analysis genotyping assay of founder animals run in duplicate. Double peaks at approximately 343bp and 351bp in length correspond to heterozygous animals, while single peaks at 351bp correspond to WT. **B)** Sanger sequencing chromatograms and alignment of WT and *Col7a1*^*del8/del8*^ rats. Displayed sequencing results from C7 Rev primer (5’-AGGCAAGATTAGGAAGGACTTGGGG-3’). WT and *Col7a1*^*del8/del8*^ chromatograms had minimal noise and evenly spaced peaks throughout. An 8bp deletion (red box) was observed in *Col7a1*^*del8/del8*^ results within exon 1. The protospacer adjacent motif (PAM) is noted (black box) and the gRNA target sequence is highlighted yellow. **C)** Predicted amino acid sequence of exon 1 revealing frameshift mutation and potential introduction of premature stop codon (*). Differences in amino acid sequence are noted within the red box.

PCR-fluorescent fragment length analysis of founder animals revealed a peak consistent throughout samples at approximately 351 bp (Fig 4A). Heterozygous founder animals were found to have an additional peak at 343 bp. Sequencing of the identified mutagenic region in *Col7a1*^*del8/del8*^ pups confirmed an 8 bp deletion in exon 1 of the *Col7a1* gene, compared to WT littermates and the Rattus norvegicus type VII alpha 1 chain (Col7a1), mRNA (Sequence ID: *NM_001106858*.*2*) on NCBI BLAST^®^ (Fig 4B). The sequencing results using the C7 reverse primer shown in Fig 4 demonstrated that the base pairs deleted are 5’-CGAGATCC-3’ (*rn6 chr8*:*117*,*694*,*643–117*,*694*,*650*), causing a frameshift mutation (Fig 4C). Furthermore, analysis of rat *Col7a1*^*del8/del8*^ litter size compared with a previously established hypomorphic mouse model maintained at the same institution demonstrated a statistically significantly larger litter size in the rat model (S1 Fig) [9].

## Discussion

To our knowledge, the creation and characterization of an RDEB rat model is the first of its kind. We identified the need to improve current models for studying RDEB due to the size and longevity constraints observed in current mouse models, which has hindered the progression and efficiency of *in vivo* RDEB research. Even in hypomorphic C7-expressing models of RDEB, two-thirds die before 28-days of age. To offer an alternative to current models, we characterized an induced 8 bp deletion in exon 1 of the *Col7a1* gene within a Lewis rat model.

The phenotype of *Col7a1*^*del8/del8*^ pups was severe, with a median survival of three days (Fig 1A). The blistered phenotype manifested primarily in the limbs of affected animals, but was also observed on the trunk, tail, mouth, pinna, and nares (Fig 1F–1H). The fragility and blistered presentation of *Col7a1*^*del8/del8*^ pups closely resembled previous RDEB mouse models [7]. Further histological analysis also displayed signs consistent with an RDEB disease state. Dermal-epidermal detachment was visualized using H&E staining (Fig 2F) and was noted in the area deep to the lamina densa using transmission electron microscopy (Fig 3). H&E staining of *Col7a1*^*del8/del8*^ skin revealed mild multifocal hyperplasia as a slightly thickened epidermis and more prominent rete pegs (Fig 2D and 2F). Additionally, the mild hypercellularity of the superficial dermis noted shortly postnatal could be indicative of an aggregation of fibroblasts and other inflammatory cells, consistent with the chronic wounding and inflammatory state that exists within RDEB [11]. These findings suggest this model adequately recapitulates RDEB phenotypically, as well as histologically.

The lamina densa is the place of origin for anchoring fibrils, which extend into or form looping structures with the sublamina densa. This ensures tight connections with underlying type I and III dermal collagen fibers [12,13]. The lamina densa of *Col7a1*^*del8/del8*^ pups was thinner, less dense, and associated with fibrous material not consistent with anchoring fibrils (Fig 3C and 3D). Additionally supported by the minimal C7 deposition in immunofluorescent staining and a lack of immunogold antibody labeling, anchoring fibrils were deemed to be absent in *Col7a1*^*del8/del8*^ tissue (Figs 2A, 2B, 3E and 3F). The absence of anchoring fibrils suggests the mechanism responsible for the observed sublamina densa split.

The characterization of this novel rat model has the potential to provide great utility for studying RDEB. Age-matched *Col7a1*^*del8/del8*^ rats provide a larger surface area than RDEB hypomorphic mice, allowing for improved ease of testing for topical and transfusion therapies (Fig 1B and 1C). The larger surface area, combined with skin that better resembles the structure and thickness of human skin, suggest that an RDEB rat model could demonstrate enhanced transferability of dermatological findings [14]. Larger litter sizes afforded by rats compared to mice were also seen as a potential benefit of modeling a recessive disorder. Given that rats have an increased surface area, larger litter sizes, and skin more structurally similar to humans, we propose that utilizing this Lewis rat model to study RDEB could help to improve study methods, efficiency, and the translational ability of novel dermatologic therapies. However, the limited survivability of *Col7a1*^*del8/del8*^ pups necessitates further investigation into life-extending measures to provide a higher efficiency of model usage. Additionally, behavioral experiments should be conducted to evaluate whether the conspecific, pro-social behavior previously reported in rats extends to improved care and nursing of diseased pups [15]. To further enhance translational ability, the creation of a rat model with a human-specific mutation should be prioritized going forward. Overall, the use of this model provides an easily maintained organism that could enable new possibilities to be explored for treating this devastating disease.

## Supporting information

S1 Fig Pairwise comparison of litter size in rat and mouse RDEB models. Rat RDEB litter size comparison to hypomorphic mouse RDEB model. Average litter size for rat RDEB was 9.00 and 5.64 for hypomorphic mouse RDEB model. Two-tailed student’s t-test. *P-value < 0.05.
(TIF)
