# Supplementary figures and images for: Creation and characterization of novel rat model for recessive dystrophic epidermolysis bullosa: Frameshift mutation of the Col7a1 gene leads to severe blistered phenotype
Source: PLoS One. 2024 May 9;19(5):e0302991. doi: 10.1371/journal.pone.0302991 (PMC11081381; doi:10.1371/journal.pone.0302991)

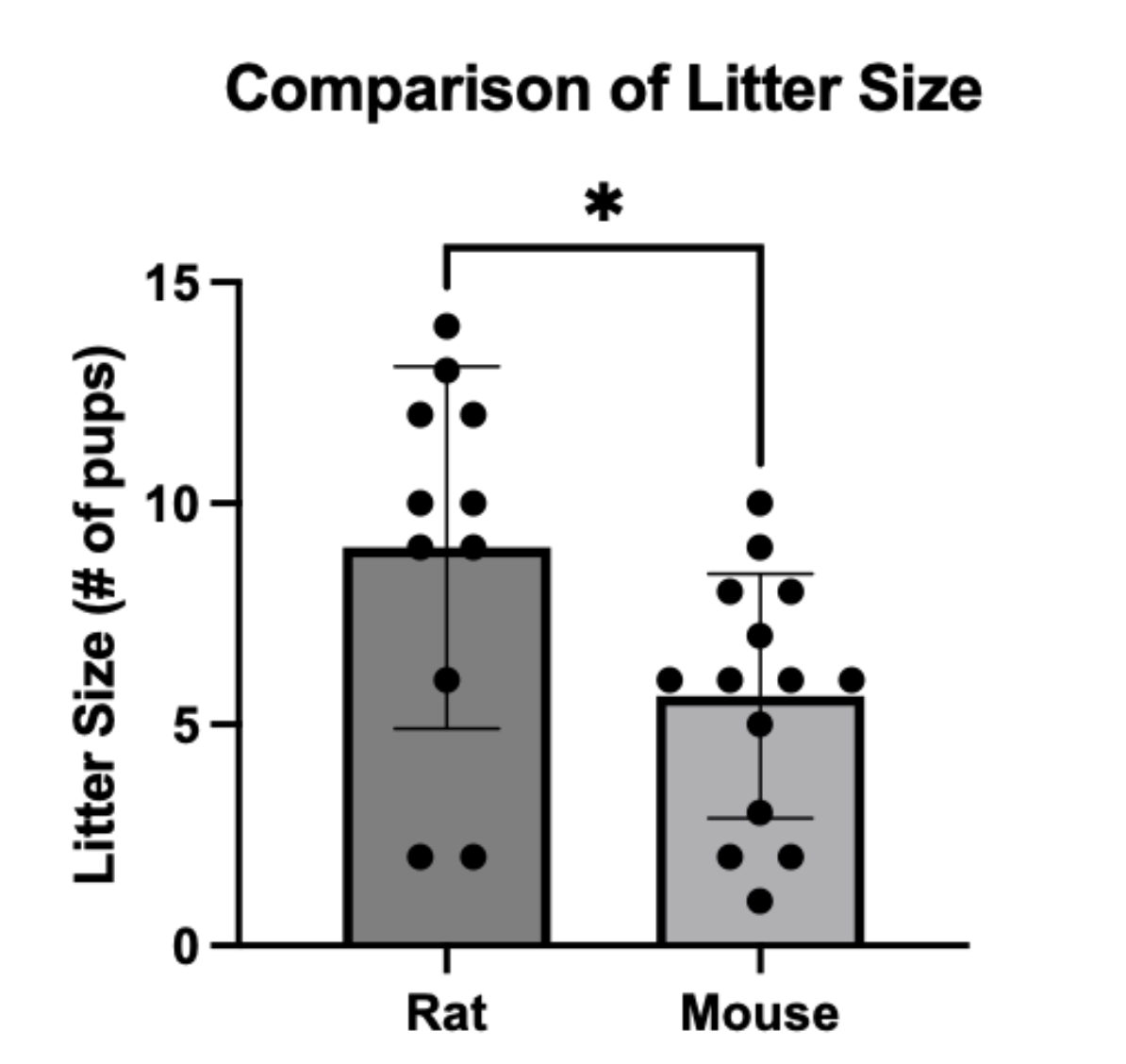

Supplement: S1 Fig — Rat RDEB litter size comparison to hypomorphic mouse RDEB model. Average litter size for rat RDEB was 9.00 and 5.64 for hypomorphic mouse RDEB model. Two-tailed student’s t-test. *P-value < 0.05. (TIF) [file pone.0302991.s001.tif]
